# Tuberculosis screening characteristics amongst freshmen in Changping District, Beijing, China

**DOI:** 10.1186/s12879-023-08802-y

**Published:** 2023-12-12

**Authors:** Xiaolong Cao, Zexuan Song, Wencong He, Zhen Yang, Qian Sun, Yiting Wang, Ping He, Bing Zhao, Zhiguo Zhang, Yanlin Zhao

**Affiliations:** 1 Chinese Center for Disease Control and Prevention, National Tuberculosis Reference Laboratory, No. 155 Chang Bai Road, Changping District, Beijing, 102206 People’s Republic of China; 2 Beijing Changping Institute for Tuberculosis Prevention and Treatment, No. 4 He Ping Street, Changping District, Beijing, 102200 People’s Republic of China

**Keywords:** Tuberculosis, Latent tuberculosis infection, Tuberculosis preventive therapy, Freshmen, Purified protein derivative

## Abstract

**Background:**

Screening for Tuberculosis (TB) is a critical tactic for minimizing the prevalence of illness within schools. Tuberculosis Preventive Therapy (TPT), in turn, effectively staves off the development of TB from latent tuberculosis infection (LTBI). Unfortunately, there is limited research on LTBI and TPT among students. This study aimed to assess LTBI among freshmen in Changping District and advocate for the implementation of TPT.

**Methods:**

The prospective study collected data from 12 educational institutions within the Changping District of Beijing. The Kolmogorov − Smirnov test and other statistical methods were used for statistical analysis, ${x}^{2}$ was obtained using the formula ${x}^{2}=$ nΣA^2^/n_R_n_C_-1, df = (C-1) (R-1). We analyzed potential factors impacting the LTBI rate, and scrutinized the possible causes behind the low application of TPT and its efficacy for LTBI treatment, China.

**Results:**

Among 19,872 freshmen included in this study, 18 active TB cases (91 per 10,0000) and 2236 LTBI cases (11.6% of 19,223) were identified, respectively. Furthermore, of those with LTBI, 1045 (5.4% of 19,223) showed a strong positive for purified protein derivative (PPD), but only 312 opted for TB preventive treatment. There appeared to be no significant difference in the prevalence of LTBI and TPT rate between male and female students. Concurrently, 11 (71 per 100,000) and 7 (158 per 100,000) cases of active tuberculosis were identified in 6 universities and 6 higher vocational colleges, respectively. Interestingly, almost all freshmen who underwent TPT came from universities, suggesting a statistically significant disparity in TPT rate (χ2 = 139.829, *P* < 0.001) between these two types of educational institutions. Meanwhile, as for the age-wise distribution of latent infection among 17–20 years old freshmen, the LTBI rate exhibited 10.5%, 11.6%, 12.1% and 13.5%, respectively. Correlation between LTBI rate, the strong positive rate was statistically significant among different ages (χ2 = 34.559, *P* < 0.001). Over a follow-up period of 2 years, three students were diagnosed with active tuberculosis, one of which was resistant to rifampicin. All three students manifested a strong positive for PPD and declined preventive treatment during TB screening.

**Conclusions:**

The data indicates a high rate of LTBI amongst students in areas with a heavy TB burden, potentially leading to cross-regional TB transmission due to the migration of students. Education level might contribute to the limited uptake of TPT. Therefore, improving the implementation of TB preventive treatments is crucial in controlling and preventing TB across schools.

## Background

Tuberculosis (TB) is the second most deadly infectious disease caused by Mycobacterium tuberculosis [1], ranking only to the COVID-19 in term of the global impact [2, 3]. Its main clinical manifestation is pulmonary tuberculosis. Due to the host's innate immunity and adaptive immunity, this bacteria can remain dormant for decades [4–6]. Approximately a quarter of the global population is estimated to be infected, with 5% to 10% of those infected developing active TB [6–8]. According to the Global Tuberculosis Report 2022, China ranks third among countries with a high TB burden. Despite various effective measures that have been implemented to reduce TB incidence, additional measures are still necessary to achieve the World Health Organization's goal of "ending TB" by 2050 [9–12]. Studies have shown that early diagnosis and intervention for treating Latent tuberculosis infection (LTBI) can help curb the development of TB. As a result, there is growing recognition that effective management of LTBI is a crucial factor in TB control [13–15]. LTBI refers to a state of persistent immune response to antigen stimulation by Mycobacterium tuberculosis, without clinical symptoms or imaging features, and without a gold standard for diagnosis [13, 16, 17]. Currently, the diagnosis of LTBI relies on the tuberculin skin test (TST) and interferon-gamma (IFN-g) release assay (IGRA). Despite limitations such as Bacilli Calmette-Guerin (BCG) cross-reactivity and potential misinterpretation results, TST remains the preferred method for mass population TB screening in high-TB burden countries due to its affordability and ease of implementation [2, 18–21].

Schools have become high-risk environments for TB transmission due to the dense population and close contact, making students and teachers particularly vulnerable to LTBI [22, 23]. The annual enrollment of freshmen in schools can be considered a mass migration, and the movement of students from high-incidence areas to schools in low-incidence areas may contribute to the spread of TB between different regions. In recent years, the incidence of tuberculosis in schools in China has significantly outpaced that in other populations, making TB control in schools as a government focal point [2]. The Chinese government has implemented the TB screening of freshmen as a crucial policy and measure into the school TB management [14, 16, 24]. The current consensus is that "intent to detect is intent to treat", the goal of TPT is to eliminate dormant bacteria. Studies have demonstrated that TPT can provide 60% to 90% protection and benefit individuals with LTBI [14, 25, 26]. However, the majority of people with LTBI across China have not yet received TPT. Previous studies have suggested that stigma of disease play a role in the failure of receive TPT, along with adverse drug reactions and a lack of TB knowledge [27, 28].

TPT has been implemented in Beijing schools, allowing students with LTBI to receive free TB drugs. Among various TPT regimens, the current recommended treatment approach is isoniazid plus rifapentine due to its minimal adverse drug reactions and high medical compliance [7, 26]. However, there is a lack of systematic studies on LTBI and the usage of TPT specifically in students. To facilitate the promotion of TPT, we plan to conduct a prospective study. The primary objective of this study is to investigate the tuberculosis screening process and analyze the characteristics of freshmen in Changping District, Beijing. Furthermore, we aimed to evaluate the effectiveness of TPT by closely monitoring the outcomes amongst students with LTBI. The finding of this study will serve as a basis for developing comprehensive strategies for TB prevention and control in the school setting.

## Materials and methods

### Study design and participants

**Fig. 1:**
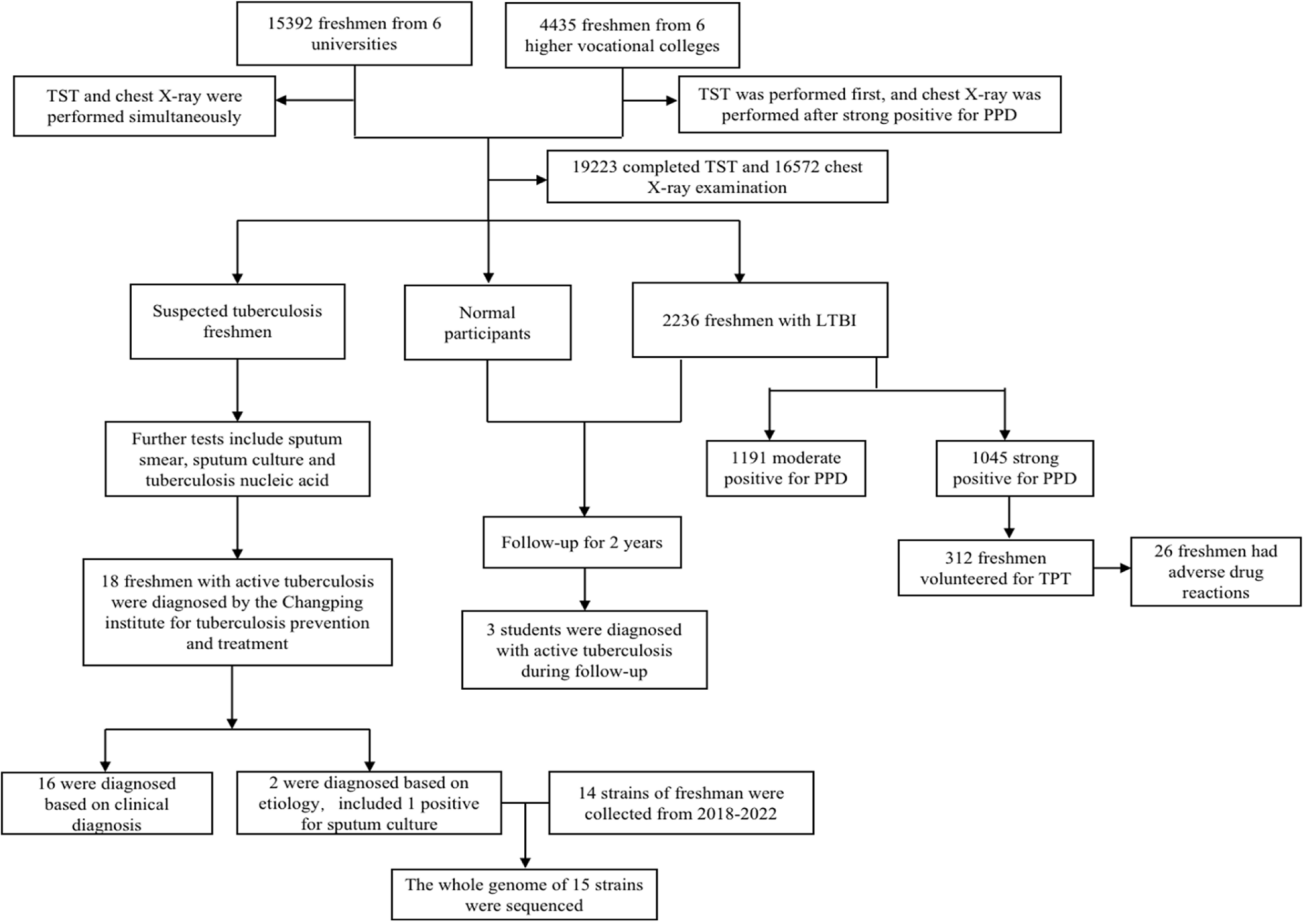
Study design and participants. TST = tuberculin skin test. LTBI = latent tuberculosis infection. TPT = tuberculosis preventive therapy. PPD = purified protein derivative

We conducted a prospective study targeting freshmen from 6 universities and 6 higher vocational colleges in Changping District, Beijing in 2020. The specific procedures are outlined in Fig. 1. TB screening was conducted from August to October 2020 and involved the use of TST and chest X-ray, the last followed-up information was collected in November 2022. The outcomes of TB Screening were categorized into three groups: suspected tuberculosis, LTBI and normal participant. Students suspected of having tuberculosis underwent further diagnosis at the Changping Institute for tuberculosis prevention and treatment. Once a student was diagnosed with active TB, standardized anti-TB treatment should be initiated. Students who tested strongly positive for purified protein derivative (PPD) were recommended to receive TPT, and followed up for 2 years to evaluate the effectiveness of the prophylaxis treatment.

### Tuberculin skin test and Chest-X-ray

Tuberculin skin test: this test involved the administration of Purified Protein Derivative of Tuberculin 20 IU (TB-PPD 20 IU) produced by Beijing Sanroad Biological Products Co., Ltd. A volume of 0.1 ml (2 IU) of TB-PPD was injected using the Montessori injection method. The injection site was observed between 48–72 h and the induration transverse and longitudinal diameters were recorded. The mean diameter was calculated as 1/2 the sum of the transverse and longitudinal diameters. According to TB diagnostic criteria (ws288-2017) in our country, PPD response ≥ 10 mm was considered as diagnostic for tuberculosis infection in areas where BCG vaccination was conducted and/or non-tuberculous mycobacterium infection was prevalent. PPD induration ≥ 5 mm or characterized by double round blister/lymphangitis, would be classified as strong positive. And TPT was administrated to students with these characteristics. TST was performed by experienced and skilled nurses from Changping Institute for tuberculosis prevention and treatment. Chest-X-ray was completed in collaboration between Changping institute for tuberculosis prevention and treatment and school hospital. All freshmen from the 6 universities were required to undergo chest X-rays. The freshmen from 6 higher vocational colleges who tested strongly positive for PPD were also requested to undergo chest x-rays, while the remaining students volunteered for the procedure [16, 27].

### Tuberculosis preventive therapy

Students who agreed to receive TPT were required to sign an informed consent form before medication, and undergo a routine liver function and blood test. The treatment regimen that consisted of isoniazid plus rifapentine for 3 months. Throughout the treatment period, regular monitoring of liver function and blood routine were conducted to observe and assess the type and severity of the adverse drug reactions. The exclusion criteria for TPT are as follows: (1) abnormal liver function, (2) refusal to sign the informed consent form for preventive medication, (3) difficulty in adhering to regular medication, and (4) other inappropriate use of medication.

### Statistical analysis

Data collection and descriptive analysis were performed using Microsoft Excel 2013. Statistical analysis was carried out using SPSS 21.0. The distribution of data was evaluated using the Kolmogorov − Smirnov test. Pearson Chi-square test and Bonferroni test or Fisher exact test were employed to compare data between groups.* P* < 0.05 was considered statistically significant.

## Results

### Tuberculosis screening of freshmen in Changping District in 2020

In this study, a total of 19,827 freshmen from 6 universities and 6 higher vocational colleges in Changping District were screened. All participants underwent at least one examination, with 19,223 (97.0%) completing the TST and 16,572 (83.6%) undergoing the chest X-ray. Among the participants, 18 cases of active tuberculosis (an incidence of 91/100000) were detected. Out of the 18 cases, 16 were diagnosed clinically, while the other 2 were diagnosed based on etiology, including 1 positive for sputum culture. Additionally, there were 2236 cases of LTBI (11.6% of 19,223) and 1045 cases of PPD positivity (5.4% of 19,223), respectively. The male participants accounted for 11,766 individuals, while the female participants numbered 8,061. Among the males, there were 10 cases of active TB (85 per 100,000) and 1306 cases of LTBI (1306/11401, 11.5%). Among the females, there were 8 cases of active TB (99 per 100,000) and 930 cases of LTBI (930/7822, 11.9%). The number of strong positive for PPD was 585 (5.1% of 11,404) among males and 460 (5.9% of 7822) among females, respectively. There was no significant difference in the incidence of LTBI and TPT rate between males and females, except for the strong positive rate (χ2 = 5.072, *P* < 0.05). The screening results were presented in Table 1.

**Table 1:** TB screening results for freshmen

	Freshmen N	TST N (%)	Chest-X-ray N (%)	Results									
				TB incidence rate	LTBI N (%)	Chi-squared test		Strong positive for PPD N (%)	Chi-squared test		TPT N (%)	Chi-squared test	
						χ^2^	*p*		χ^2^	*p*		χ^2^	*p*
Male	11,766	11,401(96.9)	9922(84.3)	10(85/100000)	1306 (11.5)	0.852	0.356	585 (5.1)	5.072	0.024	186 (31.8)	2.384	0.123
Female	8061	7822(97.0)	6650(82.5)	8(99/100000)	930 (11.9)			460 (5.9)			126 (27.4)		
Total	19,827	19,223(97.0)	16,572(83.6)	18(91/100000)	2236 (11.6)			1045 (5.4)			312 (29.9)		

*TST* Tuberculin skin test, *TB* Tuberculosis, *LTBI* Latent tuberculosis infection, *TPT* Tuberculosis preventive therapy, *N* Number, *PPD* Purified protein derivative

### Screening of universities and higher vocational colleges

A total of 6 universities (15,392) and 6 higher vocational colleges (4,435) were selected for the TB screening. The results were shown in Table 2. The TST screening rate in 6 universities was 96.9% (14,914/15392) and the chest X-ray screening rate was 100% (15,392/15392). In 6 higher vocational colleges, the TST screening rate was 97.2% (4309/4435) and the chest X-ray screening rate was 26.6% (1180/4435). There were 11 cases of active tuberculosis (71 per 100,000) in 6 universities and 7 (158 per 100,000) 6 higher vocational colleges, respectively. Among the freshmen in 6 universities, 1759 individuals (11.8% of 14,914) were diagnosed with LTBI and 785 (5.3% of 14,914) tested strongly positive for PPD. In 6 higher vocational colleges, the number of LTBI and strong positive for PPD were 477 (11.1% of 4309) and 260 (6.0% of 4309), respectively, There was a statistically significant difference in the rate of strong positive rate (χ2 = 3.859, *P* < 0.05), while there was no statistical difference in incidence of LTBI between the two types of educational institutions.

**Table 2:** TB screening results from two types of schools

	Freshmen N	TST N (%)	Chest-X-ray N (%)	Results									
				Tuberculosis	LTBI (%)	Chi-squared test		Strong positive for PPD (%)	Chi-squared test		TPT (%)	Chi-squared test	
						χ^2^	*p*		χ^2^	*p*		χ^2^	*p*
University	15,392	14,914(96.9)	15,392(100)	11(71/100000)	1759 (11.8)	1.707	0.191	785 (5.3)	3.859	0.049	210 (39.5)	139.829	< 0.001
High vocational college	4435	4309(97.2)	1180(26.6)	7(158/100000)	477 (11.1)			260 (6.0)			2 (0.8)		

*TST* Tuberculin skin test, *TB* Tuberculosis, *LTBI* Latent tuberculosis infection, *TPT* Tuberculosis preventive therapy, *N* Number, *PPD* Purified protein derivative

### Preventive treatment and follow-up

A total of 1045 (5.4%) freshmen with LTBI were strong positive for PPD. Out of these, 312 (29.9%) voluntarily opted for TPT, which included 186 males (31.8% of 585) and 126 females (27.4% of 460). In the 6 universities, 310 students with strong positive results volunteered for TPT (39.5% of 785), compared to only 2 students from the 6 higher vocational colleges (0.8% of 260). All 312 students who received TPT were followed up, 26 of them exhibited mild adverse drug reactions during treatment, common symptoms included drug-induced liver injury, leukopenia, rash. Symptomatic treatment successfully resolved these adverse effects, all students completed the preventive treatment in accordance with standard protocols. A statistical difference in TPT rate (χ2 = 139.829, *P* < 0.001) was observed between the two types of educational institutions (Table 2). 19,827 students were followed up for 2 years, 3 cases of active tuberculosis of were diagnosed, 1 of which was rifampicin-resistant TB (RR-TB), Among the 3 students, only 1 was diagnosed by etiology, while the other 2 were clinically diagnosed. All the 3 students showed strong positive for PPD during the freshmen screening, but refused to take prophylactic medication. There was no statistical difference in the incidence of TB between individuals who received TPT and those who did not receive TPT with strong positive for PPD. The details were shown in Table 3.

**Table 3:** Results of 2-year followed up of strong positive for PPD of freshmen

	Strong positive for PPD	Student with active tuberculosis were followed up for two years	Fisher exact test
	N	N	*P*
Accepted TPT	312	0	0.559
Not accepted TPT	733	3	

*N* Number, *TPT* Tuberculosis preventive therapy, *PPD* Purified protein derivative

### LTBI and ages

We categorized the freshmen who completed the TST by age, more than 1000 freshmen in the specified age group as the subjects of our analysis. The eligible age range for this analysis was 17 to 20 years old, and the respective numbers of individuals in each age group were 4412 (17), 6670 (18), 5214 (19) and 2114 (20), respectively. In the age group of 20 years, the highest rates of LTBI (13.5%, 285/2114) and strong positive for PPD (7.1%, 150/2114) were observed, respectively. For the 17, 18 and 19 groups, the LTBI rates were 10.5% (463/4412), 11.6% (774/6670) and 12.1% (631/5214), and the strong positive rate for PPD were 4.3% (189/4412), 5.1% (340/6670) and 5.7% (297/5214), respectively. The differences in LTBI rate and strong positive rate were statistically significant (*p* < 0.001) among the different age groups. Details were presented in Table 4.

**Table 4:** The results of LTBI and strong positive for PPD of freshmen between 17–20

Age	Freshmen						
	Total N	LTBI N (%)	Chi-squared test for trend		Strong positive for PPD N (%)	Chi-squared test for trend	
			χ^2^	*p*		χ^2^	*p*
17	4412	453(10.5)	16.142	< 0.001	189(4.3)	34.559	< 0.001
18	6670	774(11.6)			340(5.1)		
19	5214	631(12.1)			297(5.7)		
20	2114	285(13.5)			150(7.1)		

*N* Number, *PPD* Purified protein derivative

### LTBI among freshmen in different regions

**Fig. 2:**
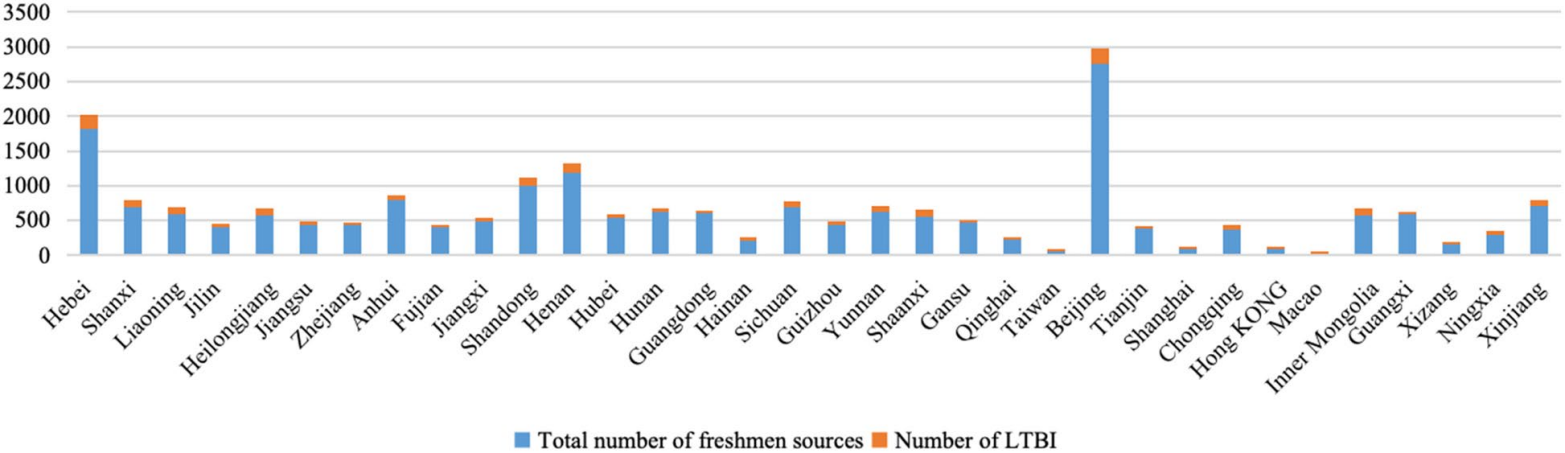
Total number of students and LTBI in each province. LTBI = latent tuberculosis infection

**Fig. 3:**
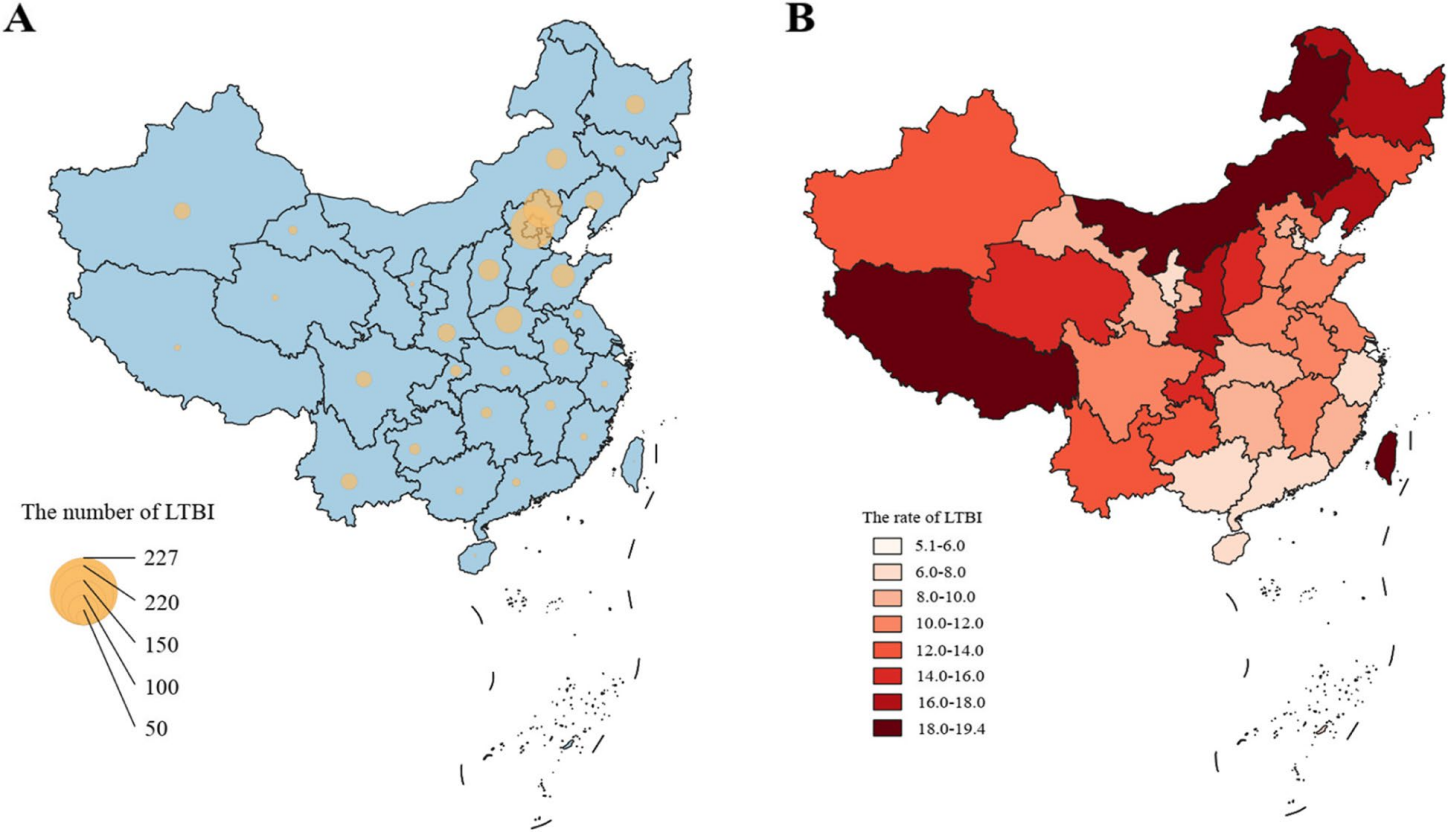
Left **A** shown the number of freshmen with LTBI by province, and right **B** shown the rate of freshmen with LTBI by province. LTBI = latent TB infection

The freshmen for TB screening came from all over the country, included 23 provinces, 4 municipalities directly under the central government, 2 special administrative regions and 5 autonomous regions. The total number of freshmen and individuals with LTBI in each province could be seen in Fig. 2. Among the 12 educational institutions, the top 6 in the number of individuals with LTBI were Beijing (227), Hebei (202), Henan (137), Shandong (119), Shanxi (109) and Inner Mongolia (107). By calculating the ratio of latent TB infection in each province, the top 6 were Xizang (32/165, 19.4%), Inner Mongolia (107/567, 18.9%), Taiwan (9/48, 18.8%), Heilongjiang (97/576, 16.8%), Shaanxi (92/555, 16.6%), Liaoning (96/585, 16.4%). The lowest LTBI rate was found in Shanghai (5/98, 5.1%). For Xinjiang, Guizhou and Qinghai, which had a high TB burden, the LTBI rates were 12.1% (86/712), 13.1% (57/434) and 15.4% (34/221), respectively. Figure 3A shown that Beijing and its surrounding provinces have the highest number of latent TB infections. As shown in Fig. 3B, latent TB infection rates were higher in Xizang, Inner Mongolia, Taiwan and Heilongjiang.

## Discussion

The school was identified as high incidence areas of TB in China [29]. Students and teachers were recognized as focus groups which were indicated by the Global Tuberculosis Report 2022. In this study, Compared previous study, the active TB screening rate of this study was 91 per 100,000 significantly higher than the TB incidence in Beijing in 2019 [30]. The incidence of TB in Beijing was lower compared with other provinces with high burden of TB, however, a large number of freshmen from various regions gather in Beijing every year, particularly from areas with a high incidence of TB. This influx of students might increase the risk of cross-regional spread of TB. Freshmen TB screening had been an essential measure for TB prevention and control in schools in China, as it effectively reduced the risk of transmission [2].

Etiological diagnosis is considered as the gold standard for tuberculosis diagnosis [31]. However, most of the 18 confirmed cases in this study were based on clinical diagnosis, which was significantly lower than the level of tuberculosis etiological diagnosis in Beijing. Two possible reasons for this low diagnosis rate are identified. First, students underwent a physical examination before the college entrance examination, which typically took about 5 months from examination to enrollment. The disease duration of TB in freshmen was short, leading to asymptomatic or mild symptoms and possibly negative sputum test results. Additionally, the quality of sputum samples was poor and subjects may not have been aware of the importance of sputum examination and failed to retain the recommended samples. Since etiological diagnosis not only contributes to diagnosing the disease, but also plays a crucial role in diagnosing the drug resistance, it is necessary to strengthen TB education of patients and conduct selective repeat sputum tests.

No statistical difference was found in TB screening rate and latent TB infection rate between different genders in this study. Previous research has shown a higher incidence of TB in males, potentially due to high-risk occupations or behaviors such as miners or smoking [32]. However, the subjects of this study were students who were not influenced by high-risk occupations or behaviors, which might explain the lack of statistical gender differences. The rate of LTBI and the strong positive for PPD were lower in freshmen compared to previous reports on other focus groups. This difference may be attributed to the screening methods, as TST was less specific than IGRA [18]. This suggests that in areas with low TB burden, IGRA can be selected for screening individuals with LTBI.

In this study, two types of educational institutions were distinguished, and the active TB screening rate of higher vocational colleges was twice that of universities. This finding suggests that higher vocational colleges should pay more attention to the tuberculosis screening of freshmen. Economic costs must be considered in countries or regions with a high TB burden and may hinder progress towards ending TB, which further emphasizes the need to optimize TB screening strategies. TST and chest X-rays were commonly used screening methods for tuberculosis in focus or high-risk groups [10, 33]. Different screening strategies were adopted for the two types of educational institutions. Through the analysis of screening results, it was found that performing TST firstly, followed by chest X-ray in cases of strongly positive for PPD, which yielded effective results in the screening of active TB or LTBI. This strategy also showed no shortcomings during the two years of follow-up. Therefore, this screening method could be adopted for large-scale TB screening to reduce the economic cost.

Between 5 and 10% of individuals with LTBI ultimately develop active TB. Therefore, the promotion of TPT plays a critical role in halting the development of TB. There is an international consensus on TPT in individuals with LTBI, and the concept of “examination intention equals treatment intention” had been proposed. Beijing has implemented a program of free medication for LTBI. Unfortunately, the rate of TPT uptake remains significantly below the target. The rate of TPT in this study was less than a third of the participants. Previous studies have identified shame about the disease and fear of adverse drug reactions as common reasons for refusing to take prophylactic drugs. Among the 312 freshmen who received TPT in this study, only 26 experienced minor adverse drug reactions that were less harmful to health and could be alleviated by symptomatic treatment. It is important to note that the harmfulness of adverse drug reactions was amplified and should not be the primary reason for rejecting TPT, except in cases with clear contraindications [28, 34]. The study also found an extremely lower rate of TPT in higher vocational colleges compared to universities, indicating that educational level might influence prophylactic medication. During the two years of follow-up, three students who shared the common characteristics of strong positive for PPD and TPT rejection were diagnosed with active tuberculosis. Although this study didn’t present a statistical difference between those who received TPT and those who did not receive TPT on the onset of TB, however, previous study has shown TPT was associated with a 32% lower incidence of active TB than placebo [35]. Other studies had shown that strong positive for PPD is a risk factor for the onset of TB, while TPT acts as a protective factor [28, 36]. TPT is an effective measure to prevent the occurrence of active TB.

The students in the study were primarily between 17 to 20 years old. In these four age groups, the rate of LTBI and strong positive rate of PPD increased with increasing age, which may be related to the decreased protective effect of BCG vaccine. Studies had shown that BCG vaccine has a good protective effect on infants and young children, but with the time, the protective effect decreases [2, 37].

The rate of the LTBI among freshmen in different provinces from 12 schools was compared and analyzed. As shown in Fig. 3A, the areas with the largest number of LTBI in this study were several provinces geographically close to Beijing, reflecting the migratory nature of the population. Figure 3B shown the rate of LTBI in different provinces, Xizang had the highest LTBI rate, as reported by a domestic study, with the incidence of tuberculosis in Xizang ranking first in the country [38, 39]. Freshmen from provinces with high TB burden such as Xinjiang, Guizhou and Qinghai also had high LTBI rates. In contrast, Shanghai had a low TB incidence, and freshmen from this city also had the lowest LTBI rate in this study [30]. This suggests that TB incidence is not well controlled in areas with high LTBI rates. In high-burden areas such as Xizang and Xinjiang, it is essential to strengthen control measures for LTBI to reduce the TB incidence. The study also observed higher latent TB infection rates among freshmen from the northeastern three provinces, indicating a vigilance need for TB incidence in the future [30]. Moreover, in addition to students, other focus or high-risk groups have not undergone standardized TB screening and TPT. Therefore, to achieve the goal of ending TB as soon as possible, addressing this issue requires in-depth consideration and solution.

## Conclusions

Screening for tuberculosis (TB) in tertiary schools has the potential to timely identify students with active TB and significantly reduce the occurrence of tuberculosis outbreaks. Our research revealed that regions with high TB burden had higher LTBI rates among students, and these rates increased with age. The migration of students also posed the risk of cross-regional spread of TB. Remarkably, less than one-third of students with strong positive for PPD underwent voluntary treatment for LTBI, with one possible explanation being the influence of education level. In this study, we did not find a statistically significant effect of LTBI treatment on reducing the TB incidence, which can be attributed to the small sample size. However, based on previous studies, we firmly believed that TPT has a definitive effect on curbing the development of TB, and its promotion would greatly contribute to the prevention and control of TB in tertiary schools.

## Data Availability

All data supporting the findings of this study are included in the article.

## Abbreviations

TB: Tuberculosis
TPT: Tuberculosis preventive therpy
LTBI: Latent tuberculosis infection
PPD: Purified protein derivative
TST: Tuberculin skin test
IGRA: Interferon-gamma release assay
